# Synergistic Radioprotection by Gamma-Tocotrienol and Pentoxifylline: Role of cAMP Signaling

**DOI:** 10.5402/2013/390379

**Published:** 2013-07-07

**Authors:** Shilpa Kulkarni, Kushal Chakraborty, K. Sree Kumar, Tzu-Cheg Kao, Martin Hauer-Jensen, Sanchita P. Ghosh

**Affiliations:** ^1^Armed Forces Radiobiology Research Institute, Uniformed Services University of the Health Sciences, Scientific Research Department, 8901 Wisconsin Avenue, Bethesda, MD 20889, USA; ^2^University of Arkansas for Medical Sciences and Central Arkansas Veterans Healthcare System, Little Rock, AR 72205, USA

## Abstract

*Purpose*. This study was designed to determine the efficacy and mechanisms of radioprotection by the combination of gamma-tocotrienol (GT3) and pentoxifylline (PTX) against acute radiation injury. *Materials and Methods*. Post-irradiation survival was monitored to determine the most efficacious dose and time of administration of PTX. Dose reduction factor (DRF) was calculated to compare the radioprotective efficacy of the combination. To determine the mechanism of synergistic radioprotection by the combination, mevalonate or calmodulin were coadministered with the GT3-PTX combination. Mevalonate was used to reverse the inhibitory effect of GT3 on 3-hydroxy-3-methyl-glutaryl-CoA reductase (HMGCR), and calmodulin was used to reverse the inhibition of phosphodiesterase (PDE) by PTX. *Results*. The combination was most effective when 200 mg/kg of PTX was administered 15 min before irradiation along with 200 mg/kg of GT3 (−24 h) and resulted in a DRF of 1.5. White blood cells and neutrophil counts showed accelerated recovery in GT3-PTX-treated groups compared to GT3. Mevalonate had no effect on the radioprotection of GT3-PTX; calmodulin abrogated the synergistic radioprotection by GT3-PTX. *Conclusion*. The mechanism of radioprotection by GT3-PTX may involve PDE inhibition.

## 1. Introduction

Exposure to lethal dose of ionizing radiation can lead to acute radiation syndrome (ARS), which is a combination of bone marrow, gastrointestinal, and cardiovascular-neuronal subsyndromes. These subsyndromes occur within hours to days based on the radiosensitivity of the tissue and the dose absorbed, and can be potentially life threatening [1]. Due to increased threat of nuclear fallout from terrorist activities and accidents at the nuclear power plants, major efforts are being invested in the development of radiation countermeasures to protect first responder military personnel and civilians [2]. Exposure to ionizing radiation is also a major concern in cancer patients undergoing radiation therapy [3]. However, no drug has been approved by Food and Drug Administration (FDA) to be used in humans against ARS.

Radiation-induced toxicity in various tissues is a manifestation of free radical generation, oxidative stress, DNA damage [4], inflammation, [5], and apoptotic signaling [6]. These signaling pathways are known to have deleterious effects in various diseases such as hypertension, diabetes, and cancer progression [5, 7]; therefore agents that have beneficial roles in modulating such signaling pathways may play important role in alleviating radiation-induced injury [8]. Various compounds such as antioxidants, thiols, antiapoptotic molecules, cytokine mimetic, and growth factors have been tested against acute radiation injury [2, 9, 10]. To determine the radioprotective efficacy of a countermeasure over a range of radiation doses, dose reduction factor (DRF) value is used. DRF is a ratio of LD_50/30_-drug over LD_50/30_-vehicle and is often used as a critical criterion used to compare efficacy of different radiation countermeasures *in vivo*. Several radiation countermeasure candidates with a DRF of 1.12 or more are being investigated for their potential use against acute radiation syndrome [11]. High DRF value is essential since it leads to protection from very high radiation doses. Our previous work on gamma-tocotrienol (GT3) [12] has shown that prophylactic treatment with GT3 24 h prior to irradiation protects mice from radiation injury with a DRF of 1.29. Radioprotection by GT3 is associated with reduction of radiation-induced DNA damage [13] and inhibition of HMGCR-mediated-nitrosative stress [14]. GT3 is also shown to increase serum interleukin-6 (IL-6) and G-CSF levels; these cytokines are known to stimulate hematopoiesis. Induction of these cytokines may contribute to radioprotective action of GT3 [15]. In an attempt to enhance the radioprotective efficacy of GT3, we tested the effect of PTX, a methyl derivative of xanthine, in combination with GT3. PTX is an FDA-approved non-specific PDE inhibitor used for intermittent claudication [16, 17]. PTX has been used alone and in combination with vitamin E (alpha-tocopherol) in preclinical and clinical studies to reduce long-term effects of radiation such as fibrosis [18–20]. Beneficial effects of PTX are contributed to its ability to inhibit proinflammatory cytokine signaling such as tumor necrosis factor-alpha (TNF-*α*) accumulation [21]. According to these studies, there was significant reduction in TNF-*α* in presence of PTX in early (2 weeks) as well as late (24 weeks) phase of radiation injury. It was recently shown that combining PTX with GT3 increased the radioprotective efficacy of GT3 in protecting mice from acute radiation injury [22]. These studies indicated that even though PTX increased the radioprotection in mice treated with GT3, its mechanism of protection was independent of endothelial nitric oxide synthase (eNOS). PTX is shown to increase nitric oxide production [23] by increasing cAMP levels. Also the effects of PTX on radiation-induced TNF-*α* levels and cAMP signaling were not evaluated. Current studies were conducted to systematically determine (a) the most efficacious time and dose of administration of PTX, (b) the DRF of the combination, and (c) the mechanisms of synergistic radioprotection by the combination. We conducted 30-day survival study to determine the most efficacious dose and time of administration of PTX. We measured the percent survival over a wide range of radiation doses to calculate the DRF of the combination. We also tested radioprotective efficacy of PTX alone. We monitored peripheral blood counts to determine the effect of GT3 and PTX on the hematopoietic system. To decipher the mechanism of synergy between GT3 and PTX, we used mevalonate to reverse the effect of HMGCR inhibition by GT3 and calmodulin to reverse phosphodiesterase inhibition, and calcium and cAMP signaling [24, 25] such as PTX. Our results indicate that the increase in the radioprotective efficacy of GT3 by combining it with PTX was due to PDE inhibition, an effect that was reversed by calmodulin administration.

We also measured lipid hydroperoxide formation (malondialdehyde) in liver microsomes to determine the effect of PTX on the ability of GT3 to inhibit lipid peroxidation. Our results indicate that increase in the radioprotective efficacy of GT3 by combining it with PTX was due to an increase in cAMP and calcium signaling, an effect that was reversed by calmodulin administration.

## 2. Materials and Methods

### 2.1. Animals

 Male CD2F1 mice (6–8 weeks old) purchased from Harlan Laboratories (Indianapolis, IN) were housed (eight per cage) at the Armed Forces Radiobiology Research Institute (AFRRI) in an air-conditioned facility accredited by the Association for Assessment and Accreditation of Laboratory Animal Care, International. Mice were maintained in air-conditioned rooms at a temperature of 21 ± 2°C with a relative humidity of 50 ± 10% and 10–15 h cycles of fresh air. The mice were quarantined for 2 weeks on arrival from the vendor. Microbiology, serology, and histopathology examination of representative samples ensured absence of *Pseudomonas aeruginosa* and common murine diseases. Mice were provided *ad libitum *a certified rodent diet (Harlan Teklad Rodent Diet no. 8604 (w), Madison, WI) and water acidified with HCl (pH 2.5–3.0). All mice were kept in rooms with a 12 h light/dark cycle with lights on from 0600 to 1800 h. All animal procedures were performed in accordance with a protocol approved by the AFRRI's Institute of Animal Care and Use Committee (IACUC). Research was conducted according to the Guide for the Care and Use of Laboratory Animals, prepared by the Institute of Laboratory Animal Resources, National Research Council, and U.S. National Academy of Sciences.

### 2.2. Irradiation

Total-body irradiation (TBI) of unanesthetized mice was performed bilaterally in well-ventilated Plexiglas boxes (partitioned for 8 mice per box) in the AFRRI ^60^Co-radiation facility at a dose rate of 0.6 Gy/min as described elsewhere [13]. ESR (electron spin resonance) dosimetry system (American Society for Testing and Material Standard E 1607) was used to measure dose rates (to water) in the cores of acrylic mouse phantoms. Phantoms were 3 inches long and 1 inch in diameter and were located in all other compartment of the exposure rack. The ESR signals were measured with a calibration curve based on standard calibration dosimeters provided by the National Institute of Standard and Technology (NIST). The accuracy of the calibration curve was verified by intercomparison with the National Physical Laboratory (NPL), United Kingdom. The only corrections applied to the dose rates in phantoms were for the decay of cobalt-60 source and for a small difference in mass energy-absorption coefficients for water and soft tissue. The radiation field was uniform within ±2%. Radiation doses for hematological studies were chosen based on our previous studies [13] conducted with the same strain of mice to ensure the availability of live animals to serve as irradiated controls for the entire experimental period.

Radiation doses for hematological studies were chosen based on our previous studies conducted with the same strain of mice to ensure the availability of live animals to serve as irradiated controls for the entire experimental period.

### 2.3. Drug Formulation and Administration

GT3 was purchased as a formulation in 5% Tween 80 in saline from Yasoo Health Inc. (Johnson City, TN). Vehicle control formulation containing an equivalent amount of olive oil in 5% Tween 80 was also purchased from Yasoo Health Inc. The final GT3 concentrations were adjusted to deliver 200 mg/kg of animal body weight in 0.1 mL formulation per animal. Control mice received 0.1 mL of vehicle. GT3 or vehicle were administered subcutaneously (SC) and aseptically (rubbing alcohol) at the nape of the neck with a 23-gauge needle, 24 h prior to irradiation. There were no local reactions observed at the site of injection. PTX, calmodulin, and mevalonate were purchased from Sigma-Aldrich (St. Louis, MO) and were formulated in saline solution to deliver 0.1 mL SC at the nape of the neck. Control mice received 0.1 mL of saline or 5% Tween 80 formulation (GT3 vehicle).

### 2.4. PTX, Mevalonate, and Calmodulin Dose and Time of Administration

Previous studies have shown that the optimum dose of GT3 was 200 mg/kg [12]. In order to determine the dose and time of administration of PTX, various treatment groups of 16 mice each were given 200 mg/kg of GT3 24 hour before, and 100 or 200 mg/kg of PTX 60, 30, or 15 minutes before 11.5 Gy total body irradiation (TBI). We also monitored 30-day survival in mice administered with PTX alone (200 mg/kg, −30 min). Mevalonate (30 mg/kg) was coadministered with GT3 24 h prior to radiation. Calmodulin was coadministered with PTX 15 min prior to radiation. There were no reports on maximum tolerated doses (MTD) of calmodulin in mice; therefore, we conducted dose escalation toxicity studies up to 30 days. Mice were injected with 2000, 5000, 10000, and 20000 units of calmodulin in saline. They were monitored daily, and weighed every other day. No toxicity was observed in all the treatment groups. 5000 unit/mouse of calmodulin was used for survival studies. Calmodulin in saline was injected along with PTX 15 min before irradiation. After irradiation, mice were returned to their cages with free access to food and water and monitored for 30 days for weight loss and survival.

### 2.5. Determination of Dose Reduction Factor (DRF)

DRF studies were performed as described elsewhere [12] using optimum dose of GT3 (200 mg/kg, −24 h). Based on the dose and time optimization studies, the following four treatment groups were used for calculation of DRF. (a) Vehicle (−24 h), (b) PTX (−15 min, 200 mg/kg), (c) GT3 (200 mg/kg, −24 h), and (d) GT3 (200 mg/kg, −24 h) plus PTX (200 mg/kg, −15 min) with 16 mice in each group. The vehicle-injected and PTX-injected groups were irradiated at 7.5, 8.0, 8.5, 9.0, 9.5, and 10.0 Gy TBI; GT3 and GT3-PTX-injected groups were irradiated at 10.5, 11.0, 11.5, 12.0, 12.5, and 13.0 Gy TBI. The range of radiation doses for vehicle or GT3-treated groups were selected based on previous observations so that the lowest radiation dose would result in 100% survival and the highest dose would result in 100% lethality. Survival was monitored for 30 days and LD_50/30_ doses for all three groups of mice were calculated using probit analysis. The DRF was calculated as the ratio of the LD_50/30_ of GT3-PTX-treated mice to the LD_50/30_ of vehicle-treated mice with 95% confidence interval.

### 2.6. Peripheral Blood Cell Counts

Mice were given one of the following treatments: (a) vehicle (−24 h), (b) PTX (100 mg/kg, −15 min), (c) GT3 (−24 h), or (d) GT3 (−24 h) and PTX (100 mg/kg, −15 min) and irradiated at 8 Gy at a dose rate of 0.6 Gy/min. This radiation dose was chosen so the vehicle-treated group would survive for the duration of the experiment (up to 60 days after irradiation). Corresponding sham-irradiated treatment groups were also used for control. Eight mice were used for each treatment group and blood was collected at 0, 1, 4, 7, 17, and 30 days after irradiation. Blood was collected (0.6 mL) from the posterior vena cava with a 23-gauge heparinized needle from mice after anesthetizing mice with isoflurane (Hospira Inc., Lake Forest, IL, USA). Blood was transferred immediately into ethylenediamine tetra-acetic acid (Sigma) containing blood collection tubes and mixed gently on a rotary shaker until analysis. Total white blood cells (WBC), absolute neutrophil counts (ANC), monocytes (MONO), lymphocytes (LYMP), platelets (PLT), and reticulocytes (RETIC) were measured using an Advia 120 cell counter (Bayer Corporation, Tarrytown, NY).

### 2.7. Bone Marrow Collection

For each treatment group, 6 mice were humanely euthanized at time points 0, 4, and 7 days post-irradiation. Femurs from 2 mice were pooled for each sample. Femurs were flushed with sterile phosphate buffer saline (PBS) containing 2% fetal bovine serum (FBS) (ATCC, Manassas, VA) using a 23 G needle. The cells were separated using a 18 G needle. Cell suspensions were filtered through a strainer and centrifuged at 400 g for 5 minutes at 4°C. Nucleated cells were counted using Beckman Coulter Counter (Beckman Coulter, Indianapolis, IN).

### 2.8. Estimation of Lipid Peroxides in Stimulated Liver Microsomes

Lipid peroxidation in stimulated liver microsomes was measured as thiobarbituric acid reactive species (TBARS). All chemicals were purchased from Sigma Aldrich. Liver microsomes were incubated with millimolar amounts of ferrous ammonium sulfate and NADPH in presence of GT3 and PTX alone and in combination at various concentrations for 15 minutes at room temperature. 10% trichloro acetic acid (TCA) was added to each sample to precipitate lipids and proteins and left on ice for 30 minutes. Samples were centrifuged for 15 minutes at 2000 g at 4 degrees. Supernatant was transferred to a glass tube. Finally 200 *μ*L of 0.67% thiobarbituric acids (TBA) was added to the supernatant and incubated in boiling water (over 90 degrees) for 10 minutes. The solutions were cooled to room temperature. Absorbance was recorded to measure amounts of TBARS at 535 nm. Known concentrations of TBA were used to generate a standard curve. Each data point was measured in triplicate with each experiment repeated twice. Percent inhibition was calculated based on TBARS species produced by NADPH in microsomes.

### 2.9. Serum Cytokine Analysis

Blood was collected (0.6 to 1.0 mL) from posterior vena cava with a 23-gauge heparinized needle from mice after deep anesthesia with isoflurane (Hospira Inc., Lake Forest, IL, USA). It was transferred immediately into serum separator tubes and allowed to clot for 30 min at room temperature. After centrifugation at 2000 g for 10 min, serum was transferred to a fresh micro-centrifuge tube and stored at −80 degrees. Serum samples were thawed and processed using Bio-Rad manufacturer's protocol. Sandwich immunoassay protocol was used to measure several cytokines (23-plex-Bio Rad) using multiplex Luminex assay. Six mice were used for each data point. The samples were analyzed using Bio-Rad's Luminex 200 instrument. 

### 2.10. Statistical Analysis

For the survival data, Fisher's exact test was used to compare % survival at 30 days with Bonferroni adjustment. The Log-rank test was used to compare survival curves. Means and standard errors were reported for peripheral blood counts. Analysis of variance (ANOVA) was used to determine if there was a significant difference among different groups. For a given day, if there was a significant difference among the groups, a pair-wise comparison was done using the Tukey-Kramer method. The significance level was set at 5% for each test. All statistical tests were two-sided. Statistical software, PC SAS, was used for statistical analyses.

## 3. Results

### 3.1. Determination of the Most Effective Time and Dose of Administration of PTX for the Radioprotective Efficacy of the GT3-PTX Combination

**Figure 1:**
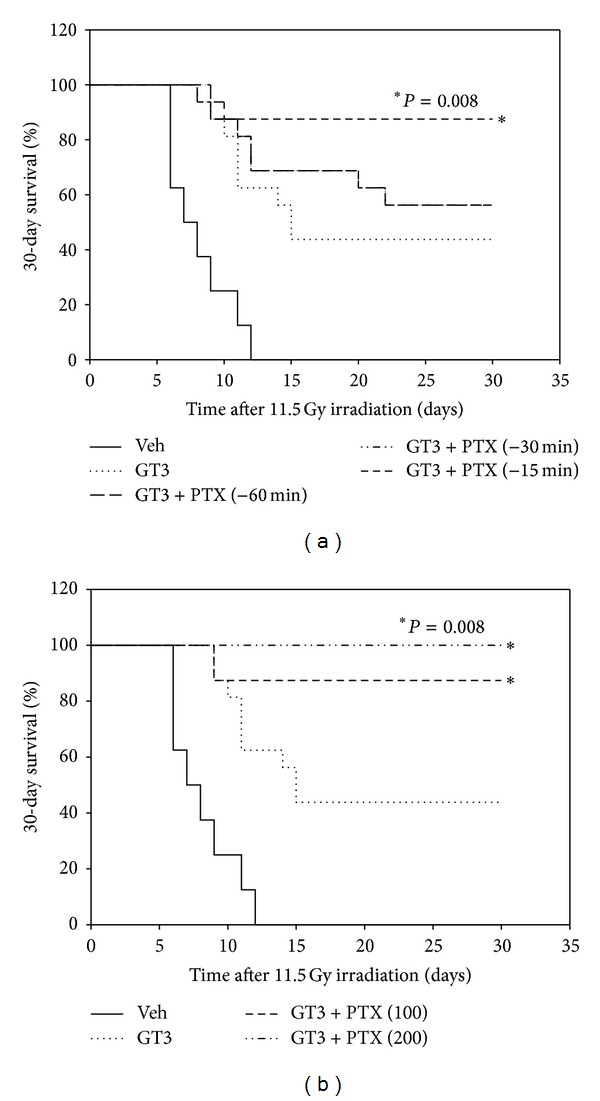
GT3-PTX combination increased the radioprotective efficacy of GT3 at 11.5 Gy. Postirradiation survival studies were conducted on mice (*n* = 16) treated with GT3 or PTX or a combination of GT3 and PTX. (a) shows time optimization studies on GT3 (200 mg/kg) and PTX (100 mg/kg) combination. GT3-PTX (−15 min) combination provided significantly greater protection than GT3 (*P = 0.008). All groups treated with GT3 alone or in combination with PTX significantly improved the survival in mice compared to vehicle (*P* = 0.0004). (b) shows that 100 mg/kg and 200 mg/kg of PTX significantly increased survival over GT3 alone (*P = 0.008). There was PTX-dose dependent increase in radioprotection but statistically it was not significant.

Time and dose of administration of PTX were chosen based on the previous studies conducted on PTX [26]. To determine the most efficacious time of administration of PTX, 100 mg/kg of PTX was administered 60 min, 30 min, or 15 min before irradiation. Percent survival (30-day) of mice treated with 200 mg/kg of GT3 (−24 h) and 100 mg/kg of PTX given at various times before 11.5 Gy irradiation is shown in Figure 1(a). The combination was most effective when PTX was given 15 min before TBI and least effective when PTX was administered 60 min before TBI. Thus administration of PTX closer to the time of irradiation was crucial for the radioprotective efficacy of the combination. Single injection of 100 or 200 mg/kg of PTX was administered to determine the most efficacious dose. Percent survival (30-day) of mice treated with 200 mg/kg of GT3 and two different doses of PTX before radiation (11.5 Gy TBI) is shown in Figure 1(b). There were no survivors in the vehicle-treated groups. 200 mg/kg and 100 mg/kg PTX in combination with GT3 resulted in 100% and 87.5% survival, respectively. Thus, the combination increased the postirradiation survival (*P* = 0.008) for both doses of PTX tested compared to the GT3 group alone. There was no significant difference between 100 and 200 mg/kg of PTX. Therefore, 200 mg/kg of PTX was used for survival studies, and 100 mg/kg of PTX was used for hematological studies.

### 3.2. Radioprotective Efficacy of PTX Alone

**Figure 2:**
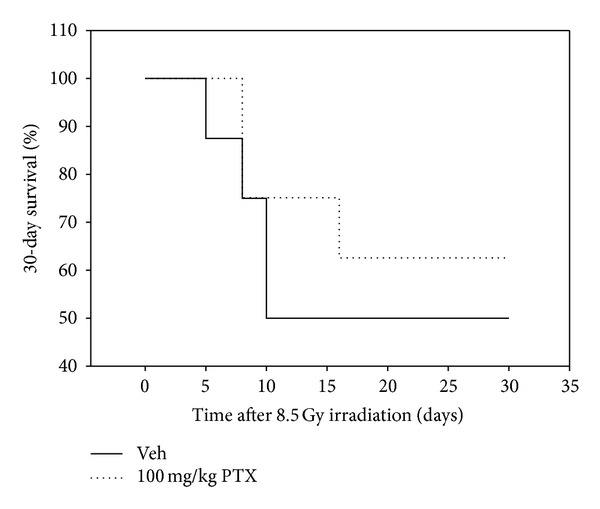
Effect of PTX alone on the postirradiation survival in mice Percent survival in mice (*n* = 16) treated with 200 mg/kg PTX or vehicle (saline) irradiated at 8.5 Gy TBI was followed for 30 days after irradiation. PTX did not increase postirradiation survival significantly, indicating that it is a poor radiation countermeasure when used alone.

To determine whether increase in radioprotective efficacy by combining PTX with GT3 was an effect, we conducted 30-day post-survival studies with PTX alone. PTX was administered 15 min before 8.5 Gy TBI, and postirradiation survival was monitored for 30 days. As shown in Figure 2, there was no significant increase in postirradiation survival with PTX alone compared to the vehicle. These studies indicate that PTX alone was a poor radiation countermeasure. Thus protective effect of GT3-PTX combination was not merely an additive effect of GT3 and PTX.

### 3.3. Determination of Dose Reduction Factor (DRF)

**Figure 3:**
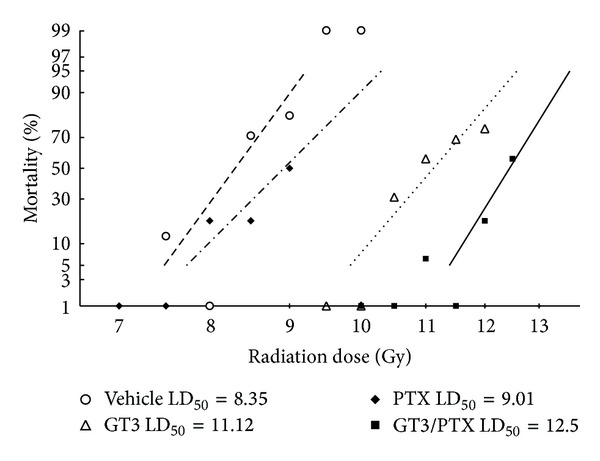
Determination of dose reduction factor for the GT3-PTX combination. Mice (*n* = 16) treated with one of the treatment groups (vehicle, 200 mg/kg of PTX, 200 mg/kg of GT3, and 200 mg/kg of GT3 plus 200 mg/kg of PTX) were exposed to a range of different radiation doses. The vehicle-injected and PTX-injected groups were irradiated at 7.5, 8.0, 8.5, 9.0, 9.5, and 10.0** **Gy TBI; GT3 group was irradiated at 9.5, 10.0, 10.5, 11.0, 11.5, and 12.0** **Gy TBI. GT3-PTX-injected groups were irradiated at 10.0, 10.5, 11.0, 11.5, 12.0, and 12.5 Gy TBI. Postirradiation survival was used to generate probit curve. Dose required for 50% mortality at 30 days (LD_50/30_) was calculated for each treatment group. Ratio of LD_50/30_-drug/LD_50/30_-vehicle, also known as dose reduction factor (DRF), was calculated as an index of radioprotective efficacy over a range of radiation doses with 95% confidence interval.

We reported earlier that the DRF for 200 mg/kg GT3 was 1.29 [12]. In order to determine the radioprotective efficacy of GT3 combined with 200 mg/kg of PTX, DRF was calculated (Figure 3) for vehicle, GT3, and the GT3-PTX combination. There was no significant difference in the LD_50/30_ radiation doses between vehicle (8.5 Gy) and PTX (9.1 Gy). LD_50/30_ doses were determined to be 11.01 (95% CI) Gy for GT3 and 12.5 (95% CI) Gy for the GT3-PTX combination. DRF of 1.5 (95% CI 1.45–1.54, Figure 3) was obtained for the GT3-PTX combination, which was significantly higher than the DRF reported for GT3.

### 3.4. Effect of the GT3-PTX Combination on Radiation-Induced Cytopenia

**Figure 4:**
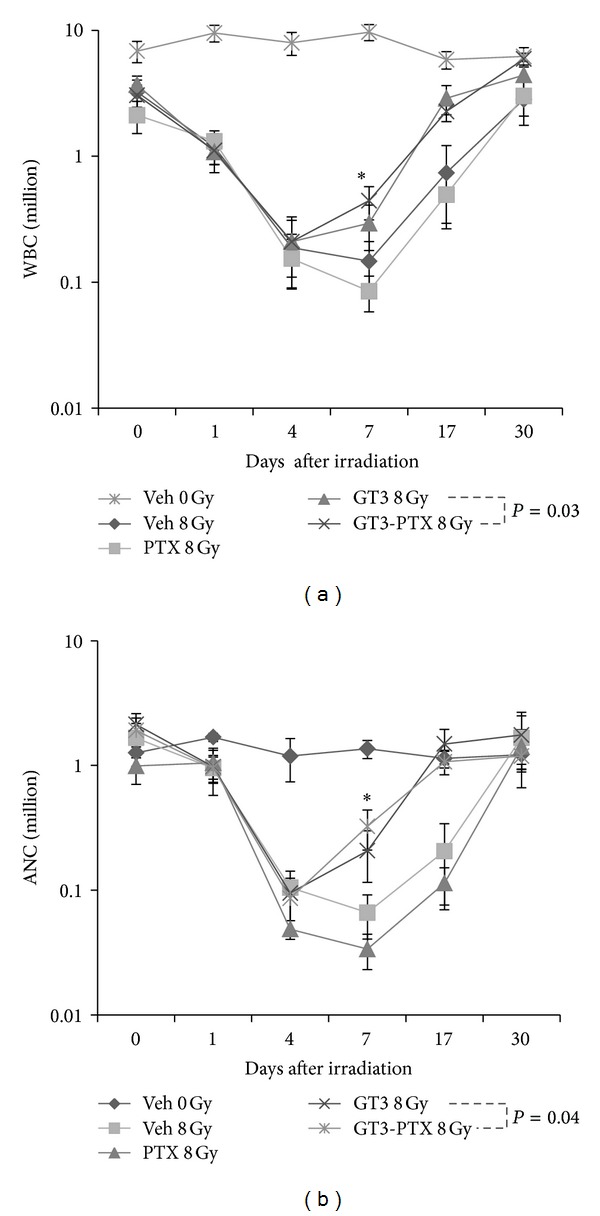
Effect of GT3-PTX combination on peripheral blood cells. Mice (*n* = 8) treated with various treatment groups (vehicle, 100** **mg/kg PTX, 200** **mg/kg GT3, 200** **mg/kg GT3 plus 100 mg/kg PTX) were exposed to 8** **Gy TBI. Corresponding unirradiated controls were also included in the experiment. (a) and (b) show WBC and ANC over a period of 30 days. GT3-PTX combination shows accelerated recovery in WBC (*P = 0.03) and ANC (*P = 0.04) at day 7. At day 30, all irradiated groups reached the normal levels.

Exposure to 8 Gy TBI resulted in rapid depletion of white blood cells (WBC), and absolute neutrophils (ANC) as observed in Figures 4(a) and 4(b), respectively. Vehicle-treated and PTX-treated groups reached nadir at day 7. There was no difference in the declination or recovery profile between irradiated vehicle- and PTX-treated groups for WBC and ANC; on the other hand, GT3 and GT3-PTX treatment groups showed accelerated recovery in WBC and ANC at days 7 and 17. WBC and ANC in GT3-PTX-treated group were significantly greater (*P* < 0.04) than GT3-treated group on day 7 at day 30 all irradiated groups showed complete recovery in WBC and ANC.

### 3.5. Effect of the GT3-PTX Combination on Bone Marrow Cells

**Figure 5:**
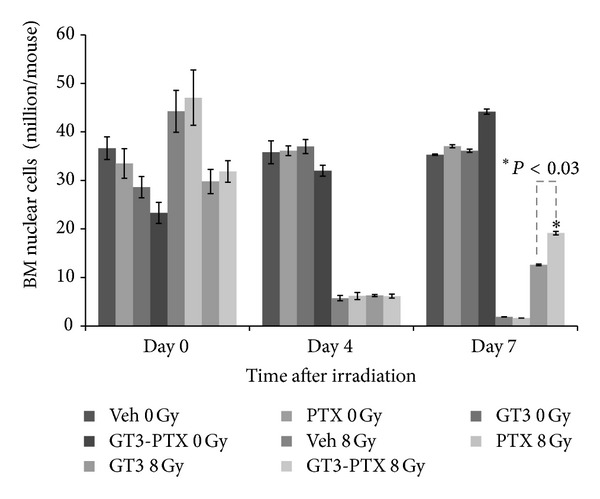
Effect of GT3-PTX on bone marrow cellularity. Bone marrow nucleated cells harvested from femurs (*n* = 6) at days 1, 4, and 7 after 8** **Gy TBI from one of the following groups: vehicle, 100** **mg/kg PTX, 200** **mg/kg GT3, 200** **mg/kg GT3 plus 100** **mg/kg PTX. Corresponding unirradiated controls were also included in the experiment. GT3-PTX combination shows significantly higher bone marrow cellularity (*P = 0.03) compared to GT3 alone group at day 7 post-TBI.

Bone marrow leukocyte population was measured in femurs to determine the hematopoietic recovery in progenitors. As shown in Figure 5, irradiation resulted in depletion of bone marrow; however, GT3- and GT3-PTX-treated groups showed remarkable recovery at day 7 compared to vehicle- and PTX-treated groups. More importantly, GT3-PTX group showed higher cellularity (*P* = 0.03) than GT3 alone group. Thus, increase in bone marrow cellularity was preceded with an equivalent recovery in peripheral blood cells.

### 3.6. Effect of GT3-PTX on Serum Cytokines and Chemokines

**Figure 6:**
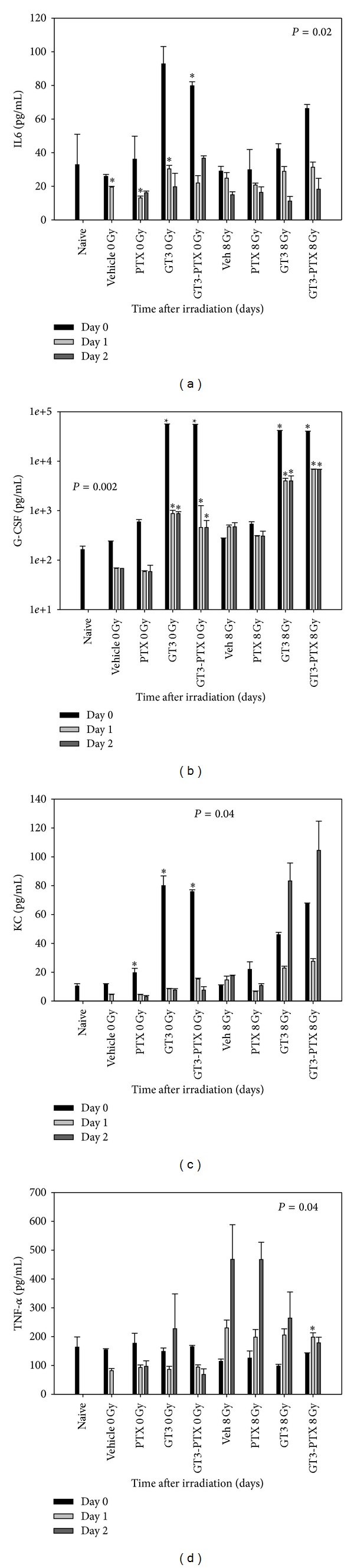
Effect of 200 mg/kg GT3 plus 100 mg/kg of PTX combination on serum cytokines. Among 21 cytokines analyzed in the serum samples, (a) interleukin 6 (IL-6), (b) granulocyte colony-stimulating factor (G-CSF), (c) keratinocyte chemoattractant (KC), and (d) tumor necrosis factor *α* (TNF-*α*) levels were altered in GT3 and GT3-PTX-treated groups. High levels of G-CSF, IL-6, and KC were observed with GT3 and GT3-PTX treatment (*P < 0.05). GT3-PTX combination synergistically decreased the levels of TNF-*α* by 3-fold compared to vehicle (*P < 0.04).

23-plex mouse cytokine assay (Bioplex, BioRad, Hercules, CA) was conducted on serum samples from mice on days 0, 1 and 2 after 8 Gy TBI. As indicated in Figure 6, granulocyte colony-stimulating factor (G-CSF), keratinocyte chemoattractant (KC), and interleukin-6 (IL-6) were upregulated and TNF-*α* was downregulated by GT3-PTX treatment. GT3 and GT3-PTX treatment upregulated IL-6 (3-fold) in the unirradiated groups, this effect disappeared in irradiated groups. G-CSF was strongly stimulated in mice treated with GT3 and GT3-PTX compared to the vehicle group. G-CSF stimulation was observed in irradiated (40,000 pg/mL) as well as unirradiated (50,000 pg/mL) groups, and the levels remained significantly high on days 0, 1, and 2 postirradiation. PTX had no effect on G-CSF levels. IL-6 levels in GT3 and GT3-PTX groups were significantly higher (90 pg/mL, *P* < 0.05) than vehicle- or PTX-treated groups only on day 0. One day after irradiation, IL-6 levels did not change significantly in irradiated groups. KC was upregulated 4-fold in mice treated with GT3 and GT3-PTX as compared to vehicle- and PTX-treated groups on days 0 and 1 after radiation. KC levels were not significantly different in irradiated groups. TNF-*α*, a proapoptotic cytokine [27] increased almost 2-fold in all treatment groups 1 day after irradiation and continued to increase (5-fold) in vehicle- and PTX-treated groups 2 days after irradiation. GT3 treatment decreased radiation-induced TNF-*α* levels to some extent; combining GT3 with PTX decreased TNF-*α* levels even further (*P* = 0.04). 

### 3.7. Effect of GT3-PTX Combination on Lipid Peroxidation

**Figure 7:**
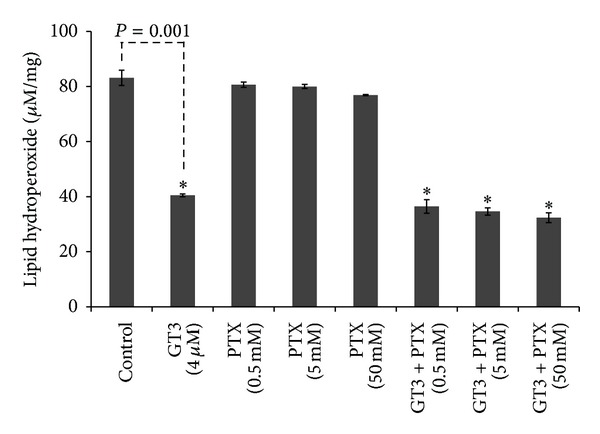
Effect of GT3-PTX combination on lipid peroxidation in liver microsomes. Lipid peroxidation was measured in millimolar amounts in the ferrous ammonium sulfate and NADPH stimulated liver microsomes incubated with various amounts of GT3 and PTX. Formation of lipid hydorperoxide, mainly malondialdehyde, was measured as a function of thiobarbituric acid reactive species per 23 mg of protein from the microsomes. GT3 containing samples show dramatic reduction in lipid peroxidation (*P = 0.001).

To determine whether PTX contributed to alleviating oxidative stress generated due to ionizing radiation, we used *in vitro* assay to measure lipid peroxidation in liver microsomes incubated with GT3 and PTX either alone or in combination. As shown in Figure 7, micromolar amount of GT3 (4 *μ*M) effectively inhibited lipid peroxidation by 50% (*P* = 0.001), whereas as much as 50 mM PTX did not inhibit lipid peroxidation. GT3-PTX combination also reduced lipid peroxidation by 50%, which was similar to the inhibition obtained with GT3 alone. Thus PTX had no effect on antioxidant activity of GT3.

### 3.8. Effect of Mevalonate on Radioprotection by the GT3-PTX Combination

**Figure 8:**
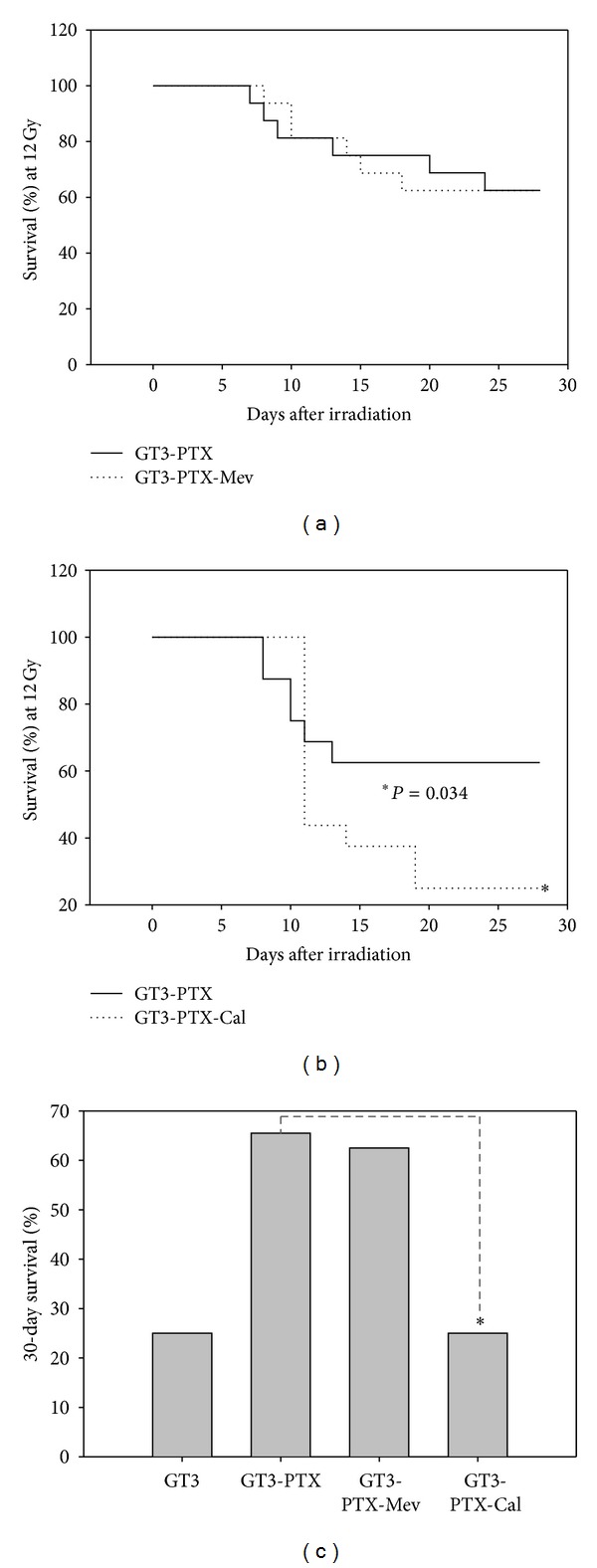
Effect of mevalonate and calmodulin on radioprotection by the GT3-PTX combination. Postirradiation survival study was conducted in mice (*n* = 16) treated with GT3, GT3-PTX, GT3-PTX-mevalonate, or GT3-PTX-calmodulin. (a) shows % survival over 30 days in mice treated with 200 mg/kg of GT3 (−24 h), 200 mg/kg of PTX (−15 min) with and without 30** **mg/kg of mevalonate (−24** **h). (b) shows % survival over 30 days in mice treated with 200** **mg/kg of GT3 (−24 h), 200** **mg/kg of PTX (−15 min) with and without 2000**** *μ*/kg of calmodulin (−15** **min). Calmodulin reduced the % survival (*P* = 0.034) when given in combination with GT3 and PTX. (c) shows the histogram of % survival in various treatment groups at the end of 30 days.

GT3 is shown to reduce vascular oxidative stress in irradiated mice through its anti3-HMGCR properties [14]. To determine the role of HMGCR inhibition in GT3-PTX synergistic protection, mice were cotreated with GT3-mevalonate and PTX prior to 12 Gy TBI. As shown in Figure 8(a), postirradiation survival in GT3-PTX (56%) and GT3-PTX-mevalonate (62.5%) groups was similar. Groups treated with the GT3-PTX and the GT3-PTX-mevalonate combination showed significantly higher survival compared to GT3 (31.25%) alone group. Thus mevalonate did not reverse the effect of GT3-PTX indicating that synergy between GT3 and PTX did not affect the mevalonate pathway.

### 3.9. Effect of Calmodulin on Radioprotection by the GT3-PTX Combination

PTX is known to increase cAMP levels by inhibition of phosphodiesterases and modulating calcium-calmodulin signaling pathways [28, 29]. To determine the effect of cAMP and calcium signaling on GT3-PTX synergistic radioprotection, mice were treated with GT3-PTX and GT3-PTX-calmodulin before 12 Gy TBI. Percent postirradiation survival was followed for 30 days and plotted in Figure 8(b). Survival in groups treated with GT3-PTX (62.5%) was significantly higher (*P* = 0.034) than groups treated with GT3-PTX-calmodulin (25%). GT3-PTX-calmodulin group showed similar survival compared to GT3 alone group indicating that calmodulin was able to reverse the synergistic radioprotective effect of the GT3-PTX combination.  Figure 8(c) is a representation of % survival at the end of 30 days postirradiation studies. GT3-PTX-calmodulin treatment show significantly lower survival compared to the GT3-PTX, and GT3-PTX-mevalonate combination.

## 4. Discussion

GT3 has shown promising radioprotective efficacy in mouse model when used 24 h prior to total body irradiation [12]. The protective effect of GT3 in preventing DNA damage and protecting stem cells [13] can in part be attributed to its free radical scavenging properties. Recent report also suggests modulation of tetrahydrobiopterin a key component of HMGCR-nitric oxide synthase (NOS) signaling by GT3 [22]. PTX has been successfully used alone and in combination with vitamin E alpha-tocopherol (AT) [18, 30] to ameliorate radiation-induced late effects such as lung fibrosis. These results indicated that GT3 protected mice from acute injury, and PTX protected from radiation-induced late injuries when used alone or in combination with AT. We hypothesized that combining PTX with GT3 may result in protection against radiation-induced acute as well late effects. It was reported earlier that combining GT3 with PTX improves the efficacy of GT3 in protecting against acute radiation injury [22]. However, this effect was observed only in the hematopoietic tissue, and these studies were conducted with 400 mg/kg of GT3, a 2-fold higher dose of GT3. Present studies were conducted to determine the optimum time and dose of administration of PTX. Plethora of literature indicated that PTX reached optimum dose in the circulation minutes after administration [31]. The efficacy of the combination was very sensitive to time of administration of PTX. We also determined the dose reduction factor, an important index used to compare efficacy of a radiation countermeasure over a range of radiation doses. DRF is used to compare the efficacy of potential radiation countermeasures in animal models [11]. DRF for GT3-PTX combination was 1.5; whereas, for GT3 it was 1.3 [12]. Thus, combining PTX with GT3 increased the LD_50/30_ value from 11.2 to 12.5, and DRF from 1.3 to 1.5, which is a significant increase in the efficacy.

Peripheral blood counts from mice exposed to 8 Gy TBI did not show any protection by PTX (100 mg/kg) alone, PTX treatment prolonged ANC nadir at 8 Gy. GT3 and the GT3-PTX combination accelerated recovery of white blood cells, and neutrophils significantly over the vehicle control in irradiated mice. The GT3-PTX combination was more effective in the recovery of WBC and ANC compared to the GT3 treatment group indicating that the recovery from cytopenia in peripheral blood was preceded by increase in bone marrow cellularity. The GT3-PTX combination also repopulated bone marrow to a greater extent at day 7 compared to GT3 treatment group. Similar results were reported with higher doses of GT3 (400 mg/kg instead of 200 mg/kg) and PTX (200 mg/kg instead of 100 mg/kg) [22]. Cytokines induced in response to GT3 treatment such as G-CSF, IL-6, and KC are known to stimulate the hematopoietic system [32]. G-CSF in particular and its related synthetic forms are used to protect against radiation-induced myelosuppression [10, 32, 33]. Recent studies show that radioprotection by GT3 may involve G-CSF induction [15, 34]. PTX had no beneficial effects on G-CSF, IL6 or KC. Only TNF-*α* was significantly reduced by GT3-PTX (*P* = 0.04). Ionizing radiation is known to induce TNF-*α* signaling, which has various pathological effects [27, 35]. An inhibitory effect of GT3-PTX on TNF-*α* may contribute to the increased overall protection from radiation injury.

To determine the underlying mechanism of this synergistic protection, we used (a) mevalonate to reverse GT3-mediated HMGCR inhibition, and (b) calmodulin to reverse PTX-induced cAMP signaling. GT3 along with delta-tocotrienol (DT3) are potent inhibitors of HMGCR, which is a rate limiting enzyme in cholesterol biosynthesis [36]. Anticancer properties of GT3 were recently shown to be mediated by its ability to inhibit the mevalonate pathway [37]. Therefore the effect of GT3-PTX on HMGCR inhibition was tested on postirradiation survival. Mice cotreated with mevalonate along with GT3-PTX combination were exposed to 12.0 Gy TBI, and postirradiation survival was monitored for 30 days. LD_50/30_ dose for the GT3-PTX combination (12 Gy) was chosen for these experiments based on the DRF studies. Survival data indicated that mevalonate did not reverse the GT3-PTX combination effects indicating that the synergistic protection offered by GT3-PTX was independent of the anti-HMGCR property of GT3. Similar studies were conducted with calmodulin. Calmodulin is known to inhibit cAMP signaling [24, 38], and reverse the effect of PDE inhibitors such as PTX. We hypothesized that administration of calmodulin may potentially inhibit synergistic effect of GT3-PTX on cAMP signaling. Cotreatment of calmodulin indeed reversed the effect of the GT3-PTX synergistic radioprotection. The reversal of protection in mice in response to calmodulin indicates that cAMP induction in presence of PTX may contribute to the synergistic radioprotection by GT3-PTX. Thrombomodulin, a membrane glycoprotein in endothelial cells that activates protein C pathway, is known to protect endothelium from radiation injury [39]. It is also known to be up-regulated in response to increase in cAMP [40]. Clinical and animal studies show that injury from ionizing radiation is associated with loss of thrombomodulin from endothelium [41]. Also, PTX is shown to increase the levels of thrombomodulin in the endothelium by increasing cAMP [40, 42]. It is conceivable that synergistic radioprotection by the GT3-PTX combination may involve beneficial effects on thrombomodulin levels.
